# N-glycosylation of ACTRIIB enhances protein stability leading to rapid cell proliferation and strong resistance to docetaxel in nasopharyngeal carcinoma

**DOI:** 10.1590/1414-431X2024e14368

**Published:** 2025-02-03

**Authors:** Qin Qin, Junfeng Li, Yinjian Shao, Lan Liu, Zhibin Luo

**Affiliations:** 1 XiangYa Changde Hospital, Changde City, Hunan Province, China

**Keywords:** N-glycosylation, ACTRIIB, Nasopharyngeal carcinoma, Docetaxel, Membrane localization

## Abstract

Nasopharyngeal carcinoma (NPC) is a malignant tumor predominantly influenced by Epstein-Barr virus infection and genetic factors. The transforming growth factor-beta (TGF-β) superfamily is implicated in various cellular processes, including tumorigenesis. This study aimed to detect the role of one TGF-β superfamily member activin receptor type IIB (ACTRIIB) in NPC. This study analyzed NPC datasets, including GSE12452, GSE102349, and GSE53819. ACTRIIB expression and N-glycosylation levels were assessed by western blot, real-time PCR, immunofluorescence, and immunohistochemistry in NPC cells and tissues. As indicated by the datasets, ACTRIIB was significantly upregulated in NPC tissues, and the up-regulation was associated with poor prognosis. This study confirmed the N-glycosylation of ACTRIIB primarily at the forty-second amino acid, an asparagine. The N-glycosylation of ACTRIIB promoted the localization of ACTRIIB to the cell membrane and prevented the degradation of the protein by lysosomes, through which ACTRIIB activated the downstream Smard1/2 to promote tumor cell proliferation and invasion. Inhibition of N-glycosylation or knockdown of ACTRIIB resulted in reduced cell proliferation and invasion and increased the cell sensitivity to docetaxel. In conclusion, N-glycosylation of ACTRIIB was a critical post-translational modification that enhanced protein stability and induced membrane localization, which facilitates the functions of ACTRIIB in cell proliferation and invasion in NPC. Inhibition of ACTRIIB N-glycosylation could potentially serve as a therapeutic strategy to improve the efficacy of chemotherapy in NPC.

## Introduction

Nasopharyngeal carcinoma (NPC) is a malignant tumor occurring in the nasopharyngeal region, the space between the back of the nasal cavity and the top of the throat. This cancer is associated with genetic mutations, environmental factors, and particularly with Epstein-Barr Virus (EBV) infection (1).

Activated receptor IIb (ACTRIIB, also named ACVR2B) is a member of the transforming growth factor β (TGFβ) family, located on the cell membrane (2). ACTRIIB transduces the activin signal from the cell surface to the cytoplasm thus regulating many physiological and pathological processes including cell cycling, wound healing, extracellular matrix production, immunosuppression, and carcinogenesis (3,4). ACTRIIB plays a complex role in the development and progression of cancer, as it can affect tumor growth, invasion, and metastasis through multiple mechanisms (3,5,6). Specifically in NPC, the function of ACTRIIB and its potential therapeutic value have attracted the attention of researchers (7). The TGF-β signaling pathway involving ACTRIIB has been shown to be related to tumor growth, invasion, and metastasis in various cancers (7,8). In addition, ACTRIIB may promote the cross-talk of tumor with micro-environment, thereby affecting both tumor cell behavior and the tumor micro-environment (8-10). The TGF-β signaling pathway also plays a role in tumor immune escape (11). It can inhibit the activity and proliferation of T cells, promoting tumor immune escape (11). As a key component of this pathway, ACTRIIB may play a significant role in the regulation of immune response of T cells to NPC (12).

Multiple protein post-translational modifications (PTMs) have been found, including phosphorylation, glycosylation, ubiquitination, nitrosylation, methylation, acetylation, among others (13). Glycosylation, one of the major types of protein post-translational modifications, significantly impacts protein folding, conformation, distribution, stability, and activity (14). Glycosylation encompasses a range of options for the addition of sugar moieties to proteins, from simple monosaccharide modifications of nuclear transcription factors to highly complex branched polysaccharide variations on cell surface receptors (15). N-linked oligosaccharides attaching to asparagine (Asn) are major structural components of many cell surface and secreted proteins (16). N-glycosylation is a highly conserved and common post-translational modification, particularly for transmembrane proteins (17). As indicated by public dataset websites such as Uniprot (https://www.uniprot.org/) and NetNGlyc-1.0 (https://services.healthtech.dtu.dk/services/NetNGlyc-1.0/), three potential Asn glycosylation sites, ACTRIIB-N42, -N65, and -N486, have been found on ACTRIIB. As N-glycosylation generally confers profound impact on protein functions, this study aimed to identify ACTRIIB N-glycosylation and explore its functions in NPC.

Concurrent chemoradiotherapy has been established as the standard treatment for locally advanced NPC, significantly reducing the risk of local recurrence and distant metastasis (18,19). We utilized the NPC dataset GSE102349 (comprising 113 tumor tissues) for drug sensitivity prediction. Drug sensitivity was predicted for all patients using the Cancer Drug Sensitivity Genomics (GDSC) database (https://www.cancerrxgene.org/) and the R package oncoPredict with the “pRRophetic” algorithm, estimating the half-maximal inhibitory concentration (IC50) (20). The analysis showed that the IC50 for docetaxel is significantly increased in the ACTRIIB-highly-expressed group, suggesting that high expression of ACTRIIB may lead to the resistance of NPC to docetaxel. Docetaxel is a cytotoxic agent that induces sustained mitotic blockade, followed by apoptosis (21,22). This study also investigated the effect of ACTRIIB N-glycosylation on the sensitivity of NPC cells to docetaxel.

## Material and Methods

### Analysis of NPC datasets

The NPC datasets GSE12452 and GSE53819, both of which include normal nasopharyngeal tissue and NPC tissues, were selected. The GSE12452 dataset comprises 41 samples: 10 normal and 31 tumor samples, whereas the GSE53819 dataset includes 36 samples: 18 normal and 18 tumor samples. This study analyzed the differentially expressed genes (DEGs) between NPC tissues and non-cancerous benign nasopharyngeal tissues. DEGs selection criterion was: |logFC| > 0.5, P<0.05. Whether DEGs are associated to the prognosis of patients with NPC was determined by analyzing dataset GSE102349, which contains 88 NPC samples with recurrence-free survival (RFS) prognosis (excluding samples without prognosis and those unsuitable for prognostic analysis). We utilized the NPC dataset GSE102349 (comprising 113 tumor tissues) for drug sensitivity prediction. Based on ACTRIIB expression levels, the samples were divided into two groups (ACTRIIB-high and ACTRIIB-low) using the median value. Drug sensitivity was predicted for all patients using the Cancer Drug Sensitivity Genomics (GDSC) database (https://www.cancerrxgene.org/) and the R package oncoPredict with the “pRRophetic” algorithm, estimating the IC50.

### Western blot

Approximately 0.5 mg of tissue and cell samples were placed in a tissue homogenizer with 1 mL of Total Protein Extraction Kit (ProMab, Cat. No. SJ-200501, China). The samples were homogenized for 5-20 min until the tissue was fully broken down. Antigen (tissue/cell lysate) samples were then loaded into gels for SDS-PAGE electrophoresis. The gel composition was: 30% acrylamide solution, 10% ammonium persulfate, TEMED (Shanghai Sangon, China), and Tris-Base, 10% SDS (SIGMA, USA). The gel was then prepared for transfer by soaking in electrophoresis transfer buffer for 15-20 min. The composition of the electrophoresis buffer was: 3 g Tris-Base, 14.4 g glycine, 1 g SDS, with distilled water added to 1 L, and a pH of about 8.3. The transfer buffer composition was: 3 g Tris-Base, 14.4 g glycine, 200 mL methanol, with distilled water added to 1 L. After blocking, the membrane was washed 2-3 times with phosphate-buffered saline plus Tween (PBST) and incubated with primary antibodies (anti-ACTRIIB antibody, 1:400 Biorbyt (UK), orb1281502; anti-Smad1 antibody, 1:100, Abcam (UK), ab53745; anti-p-Smad1 antibody, 1:500 Abcam, ab226821; anti-Smad2 antibody, 1:500 Abcam, ab33875; anti-p-Smad2 antibody, 1:500 Abcam, ab280888; anti-HA antibody, 1:2000 Ptgcn, 51064-2-AP; and anti-β-actin antibody, 1:2000, Ptgcn, 66009-1-Ig) on a shaker at room temperature for 2 h. The primary antibody was then discarded, and each well was washed with 2-3 mL PBST on a shaker for 5-10 min, repeated four times. Each well then received approximately 1 mL of HRP-conjugated secondary antibody (Goat Anti Rabbit IgG/HRP, 1:4000; Goat Anti Mouse IgG/HRP,1:4000; Abcam) before incubation overnight at 4°C. The next day, the membrane was washed four times with PBST. Finally, an appropriate amount of luminescent substrate reagent was added to each membrane, sufficient to soak the strips. Chemical luminescence was then performed as soon as possible to obtain the film.

### Real-time PCR

First, RNA extraction was performed by taking approximately 30 mg of each sample, thoroughly grinding it with a homogenizer, and then adding 1 mL of Trizol. The supernatant was discarded, and the RNA pellet was washed with 1 mL of 75% ethanol, mixed well on a vortex, and then centrifuged at 7,500 *g* for 5 min at 2-8°C. The diluted and mixed RNA sample was added to the cuvette to measure the A260 value. After discarding the RNA sample from the cuvette and rinsing with ddH_2_O, the A280 zero point was adjusted, and the A280 value of the RNA sample was measured again. The A260/A280 ratio was calculated with a ratio ≥1.8 meeting the experimental requirements. Subsequent steps included the removal of genomic DNA by sequentially adding the following reagents: total RNA 1 µg, 10× reaction buffer 1 µL, RiboLock™ (Thermo Fisher Scientific) RNase Inhibitor (40 U/µL) 0.25 µL, DEPC-treated water up to 9 µL, DNase I (1 U/µL) 1 µL, and incubating at 37°C for 30 min; then, 1 µL of 25 mM EDTA was added, and the mixture was incubated at 65°C for 10 min to inactivate the enzyme before reverse transcription. After the cycle ended, melt curve analysis was performed, measuring between 60-95°C with a temperature increase of 0.5°C per step, 5 s per step. The PCR primers used were: ACTRIIB-F: GCAACTTCTGCAACGAACGC; ACTRIIB-R: GCGATGCCGGTACATCCAAA; β-actin-F: CATTAAGGAGAAGCTGTGCT; β-actin-R: GTTGAAGGTAGTTTCGTGGA.

### Cell proliferation assay

The cell proliferation capability was assessed using the Cell Counting Kit-8 (CCK-8) from Beyotime (China, #C0038). For the experiment, approximately 5×10^3^ cells per well were seeded onto a 96-well plate, with the culture system volume adjusted to 100 µL. The experimental setup included three replicate wells for each group. A background control well (complete medium with CCK-8 solution, without cells) was also set up. After culturing for the predetermined time, 10 µ/L of CCK-8 solution was added to each well, followed by incubation in a CO_2_ incubator for 1 h. The 96-well plate was then placed on a shaker and shaken for one minute to ensure thorough mixing of the test system. The absorbance was measured at 450 nm. Cell proliferation rate calculation was: Cell proliferation rate % = (absorbance value of the test well - absorbance value of the background control well) / (absorbance value of the control cells - absorbance value of the background control) × 100%.

### Cell invasion assay

First, Transwell chambers were prepared with a coating of the basement membrane: the upper surface of the Transwell chamber membrane was coated with a 1:8 dilution of 50 mg/L Matrigel and air-dried at 4°C. The basement membrane was then hydrated by adding 60-80 µL of the diluted Matrigel (3.9 µg/µL) onto the upper chamber's polycarbonate membrane (care should be taken not to use excessive volume; just enough to moisten the polycarbonate membrane is optimal), and incubated at 37°C for 30 min to allow the Matrigel to polymerize into a gel. Subsequently, cell suspension was prepared by digesting cells, centrifuging to remove the culture medium after stopping the digestion, and washing 1-2 times with PBS. Cells were resuspended in serum-free medium containing BSA. The cell density was adjusted to 5×10^4^/mL. Cells were seeded onto the chamber, with 1 mL of medium containing FBS added to the lower chamber of a 6-well plate. The cell suspension was added to the upper chamber and cultured for the set duration. Afterwards, the basement membrane in the lower chamber was retrieved. To analyze the results, a cotton swab was used to remove the matrix gel and cells inside the upper chamber, followed by fixation with 95% alcohol for 15-20 min, and staining with hematoxylin for 10 min. Finally, the samples were observed and photographed under an inverted microscope (TCS-SP5, Leica, Germany) at 200× magnification.

### Cell scratch assay

A colored marker was used to draw straight lines (reference lines) on the back of a 24-well plate using a ruler as a guide. Approximately 1×10^5^ cells/mL were added to each well, setting up different groups and interventions as required for the experiment. The exact number of cells should allow for the cells to grow to cover more than 90% of the well bottom by the second day. After the cells have reached confluence, a 20 µL pipette tip was used to scratch the surface and make a straight line across the center of the cell layer in each well, following the previously drawn reference lines. The scratches were photographed immediately as the 0-h control. Each well was washed three times with serum-free medium to remove the detached cells, and then the plate was incubated at 37°C with 5% CO_2_. At the set time point for the experiment, the 24-well plate was photographed for analysis.

### Immunofluorescence

Cells on 6-well plates were removed and rinsed with 1× PBS for 5 min. The cell slides were then fixed in fixative for 20 min and air-dried before being washed with 1× PBS buffer solution (0.01 M, pH 7.2) for 3∼5 min. Normal goat serum blocking solution was added, and the slides were incubated at room temperature for 20 min. Excess liquid was discarded. Primary antibody (50 μL of rabbit polyclonal ACVR2B antibody, 1:100 dilution, Biorbyt, orb1281502) was applied, and the slides were incubated overnight at 4°C. After washing three times with PBS for 5 min each, fluorescently labeled secondary antibody (50 μL of goat anti-rabbit IgG (H+L) diluted 1:100 (green), Alexa Fluor 488, A32731) was added, and the slides were incubated at 37°C for 40 min. This step was repeated, followed by three PBS washes, each for 5 min. DAPI was used to restain the cell nuclei for 5 min, followed by three more PBS washes. Finally, observations and recordings were made using a Leica TCS-SP5 laser scanning confocal microscope.

### Flow cytometry detection of cell apoptosis

This study used the kit for Annexin V-PE/7-AAD-staining apoptosis detection (Catalog Number KA3809, 100 assays; Abnova, China). The kit utilizes the red fluorescent dye PE (phycoerythrin)-labeled Annexin V as a probe to detect the occurrence of early apoptosis, which can be detected using a fluorescence microscope, flow cytometer, or other fluorescence detection equipment. The 7-AAD provided in this kit can be used to distinguish between viable early cells and necrotic or late apoptotic cells. 7-AAD is a nucleic acid dye with fluorescence characteristics similar to PI, but with a narrower emission spectrum, causing less interference with other detection channels, making it the best substitute for PI in multicolor fluorescence analysis. It can be used in conjunction with Annexin V. This dye cannot penetrate the intact cell membrane of normal cells or early apoptotic cells but can penetrate the membrane of late apoptotic or necrotic cells and bind to their DNA. Therefore, when Annexin V-PE and 7-AAD are used together, 7-AAD is excluded from living cells (Annexin V-/7-AAD-) and early apoptotic cells (Annexin V+/7-AAD-), while late apoptotic and necrotic cells are double-stained with both Annexin V-PE and 7-AAD, showing double positivity (Annexin V+/7-AAD+).

### Clinical study

This study collected 10 NPC and 10 benign nasopharyngeal tissue samples from XiangYa Changde Hospital. The clinical study was approved by the Ethics Committee of Xiangya Changde Hospital (approval number: 20249). Written informed consent for publication was obtained from all participants. The clinical samples were subjected to WB and PCR to detect the expression of ACTRIIB; IHC was used to examine the expression and location of ACTRIIB in NPC and benign nasopharyngeal tissues.

### Protein site-directed mutagenesis experiment

Site-directed mutagenesis, also known as point mutation or site-specific mutagenesis, is a crucial experimental technique used for studying protein function, structure, and protein-protein interactions. Initially, the protein sites to be mutated were determined based on prior bioinformatics analysis and structural information. A pair of complementary oligonucleotide primers were designed, containing the desired mutations at the mutation sites. The sequences used were for ACTRIIB-F GCAACTTCTGCAACGAACGC; ACTRIIB-R GCGATGCCGGTACATCCAAA; β-actin-F CATTAAGGAGAAGCTGTGCT; β-actin-R GTTGAAGGTAGTTTCGTGGA.

PCR was performed using the designed primers and an appropriate DNA template to introduce the specific mutations. After the PCR reaction, the amplified DNA fragments needed to be purified to remove unreacted primers and dNTPs. This was followed by plasmid construction and selection and verification, using DNA sequencing to verify the mutation sequence in the selected clones, ensuring that the correct mutation had been introduced.

### Statistical analysis

Statistical analysis was performed using SPSS 23.0 software (IBM, USA) and GraphPad Prism 7.0 (USA). For comparison between two groups, an unpaired *t*-test was performed. For comparison among three or more groups, an ordinary one-way ANOVA test was used. The results are reported as means±SE. Statistically significant differences were considered when P<0.05.

## Results

### ACTRIIB was increased in NPC and the high expression was associated to poor prognosis

**Figure 1:**
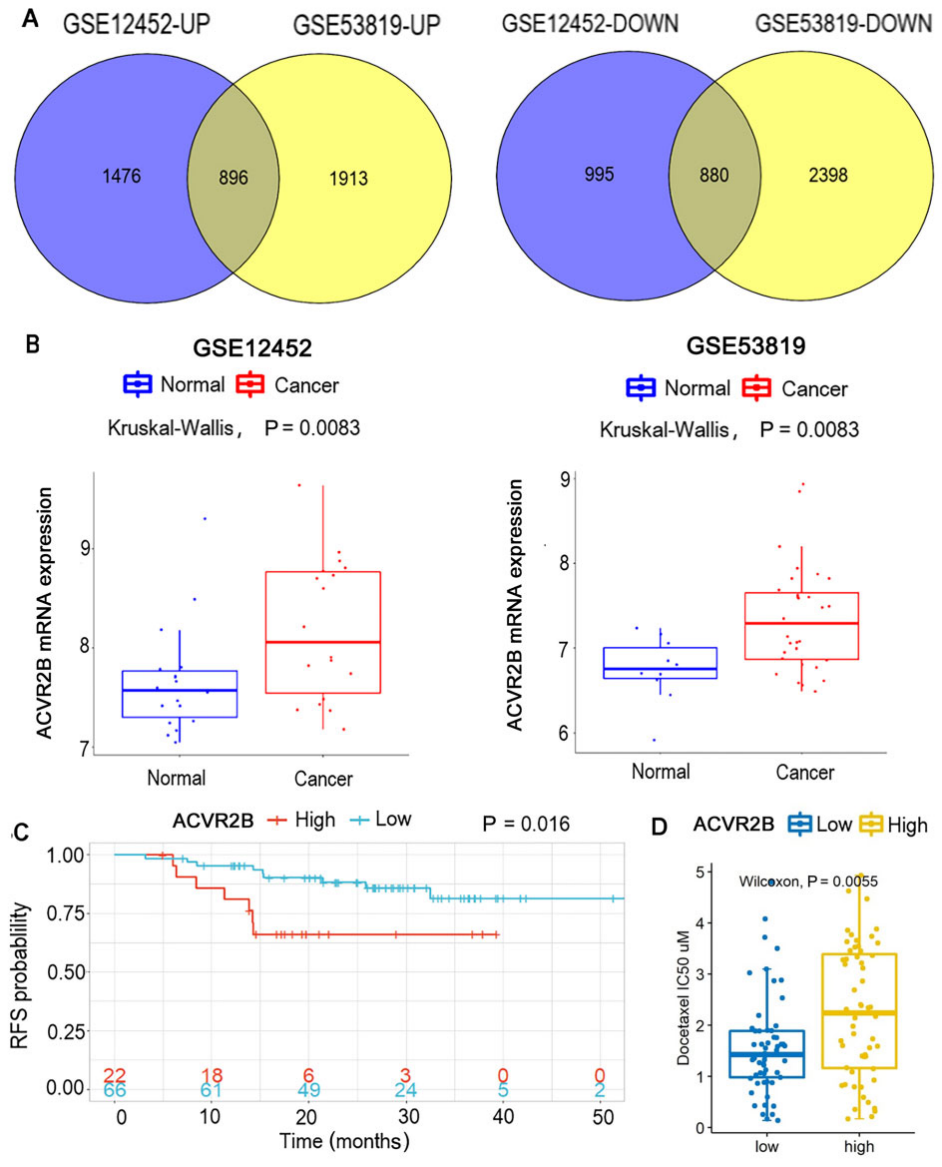
Bioinformatics analysis showed that ACTRIIB was increased in nasopharyngeal carcinoma (NPC) and the high expression was associated to poor prognosis. **A**, Intersection analysis of datasets GSE12452 and GSE53819; **B**, Expression of ACTRIIB in normal and tumor tissues according to datasets GSE12452 and GSE53819; **C**, High expression of ACTRIIB in cancer tissues of NPC was associated with poor prognosis. **D**, As indicated by GDSC dataset, high expression of ACTRIIB was associated with increased IC50 of docetaxel for tumor therapy.

Intersection analysis of datasets GSE12452 and GSE53819 revealed that 896 genes were upregulated and 880 genes were downregulated in NPC (Figure 1A). Among the up-regulated genes, ACTRIIB caught our attention for the following reasons. First, ACTRIIB expression was increased in NPC according to datasets GSE12452 and GSE53819 (Figure 1B). Secondly, high expression of ACTRIIB was associated with poor RFS according to dataset GSE102349 (Figure 1C). Third, as indicated by GDSC dataset, high expression of ACTRIIB was associated with increased IC50 of docetaxel and other drugs for tumor therapy (Figure 1D and Table 1). This suggested that ACTRIIB regulated the sensitivity of NPC to these drugs. In addition, increasing studies have showed that many members of the TGF family, including ACTRIIB, can promote tumor growth, invasion, and metastasis (3,5,6).

**Table 1:** Top ten drugs or small molecules that the half-maximal inhibitory concentration (IC50) was associated to ACTRIIB expression.

Drug ID	Low risk (mean)	High risk (mean)	P value	log2 diff (high *vs* low)
Docetaxel_1819	0.538	3.778	0.025	2.811944624
Vinorelbine_2048	0.107	0.404	0.433	1.916744496
Luminespib_1559	0.16	0.581	0.443	1.860466259
Vincristine_1818	0.364	1.311	0.072	1.84865733
Teniposide_1809	2.284	7.132	0.471	1.642744052
Gemcitabine_1190	1.667	4.983	0.69	1.57976047
Mitoxantrone_1810	1.895	5.268	0.055	1.475057497
Daporinad_1248	0.026	0.062	0.262	1.253756592
PRIMA-1MET_1131	91.196	175.616	0	0.94538184

### Knockdown of ACTRIIB suppressed the proliferation, migration, and invasion of NPC cells

**Figure 2:**
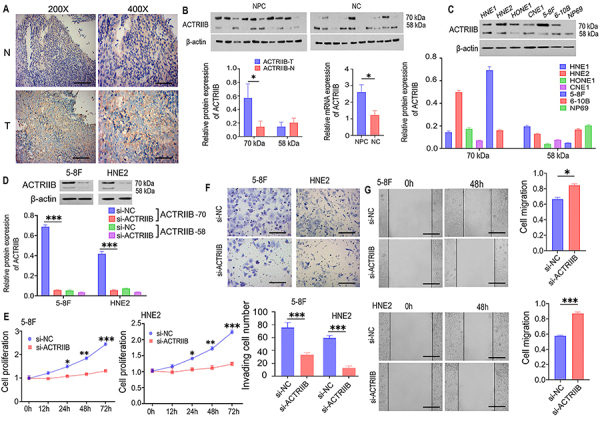
Knockdown of ACTRIIB suppressed the proliferation, migration, and invasion of nasopharyngeal carcinoma (NPC) cells. **A**, IHC detection of ACTRIIB expression in NPC and non-cancerous benign nasopharyngeal tissues. Scale bars in the left images indicate 100 μm and in the right images, 40 μm. **B**, WB and PCR detection of ACTRIIB expression. **C**, Expression of ACTRIIB protein in NPC cell lines, including HNE1, HNE2, HONE1 (poorly differentiated squamous carcinoma), CNE1 (well-differentiated squamous carcinoma), 5-8F (poorly differentiated with high metastasis), and 6-10B (low metastasis) and immortalized nasopharyngeal epithelial cells NP69. **D**, WB verification of si-ACTRIIB knockdown efficiency. **E**, Effect of si-ACTRIIB on NPC cell proliferation. **F**, Effect of si-ACTRIIB on NPC cell invasion. Scale bars indicate 40 μm. **G**, Effect of si-ACTRIIB on NPC cell scratch healing ability. Scale bars indicate 100 μm. Data are reported as means and SD (n=3). *P<0.05, **P<0.01, and ***P<0.001; *t*-test and ANOVA. NC: negative control.

ACTRIIB expression was up-regulated and primarily located in the cell membrane in tumor tissues, while in benign tissues, it was lower and mainly located in the cytoplasm (Figure 2A). The results of WB and PCR indicated that the overall expression of ACTRIIB was higher in NPC than in benign tissues. ACTRIIB protein analysis showed two bands with molecular weights of 58 kDa and 70 kDa. The higher molecular weight band was likely an N-glycosylated modification band, which was significantly more prominent in tumor tissues (P=0.036, Figure 2B).

The expression of the ACTRIIB protein was also detected in NPC cell lines, including HNE1, HNE2, HONE1 (poorly differentiated squamous carcinoma), CNE1 (well-differentiated squamous carcinoma), 5-8F (poorly differentiated with high metastasis), and 6-10B (low metastasis), and immortalized nasopharyngeal epithelial cells NP69. The results revealed that ACTRIIB expression was higher in 5-8F and HNE2, with more abundance at the high molecular weight (70 kDa), compared to NP69 cells. NP69 cells had the lowest expression without the high molecular weight form, only presenting the low molecular weight form (58 kDa) (Figure 2C). Based on WB results, 5-8F and HNE2 were selected for subsequent experiments to verify whether si-ACTRIIB inhibited NPC cell proliferation. ACTRIIB was detected through WB and PCR followed by assays for cell proliferation, wound healing, and cell invasion. The results showed that knockdown of ACTRIIB decreased ACTRIIB protein levels, especially the protein of high molecular weight (Figure 2D). Knockdown of ACTRIIB suppressed cell proliferation, invasion, and wound healing abilities in 5-8F cells and HNE2 cells (Figure 2E-G).

### N-glycosylation modification was at N42 of ACTRIIB protein in NPC cells

**Figure 3:**
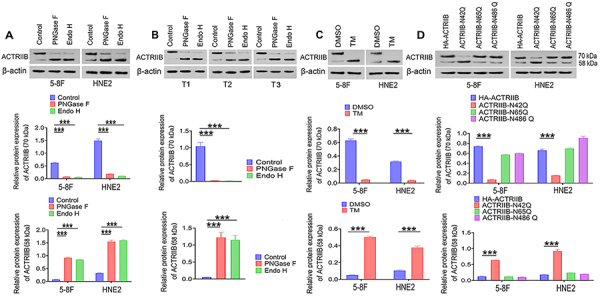
N-glycosylation modification was at N42 of ACTRIIB protein in nasopharyngeal carcinoma (NPC) cells. **A**, Removal of N-glycans from protein in 5-8F and HNE2 cells using PNGase F and Endo H, followed by WB detection. **B**, Removal of N-glycans from protein in tumor samples using PNGase F and Endo H, followed by WB detection. T1, T2, and T3: Tumor samples 1, 2, and 3. **C**, WB detection of bands of ACTRIIB protein after pre-treatment of cells with the N-glycosylation inhibitor tunicamycin (TM). **D**, The wild type (HA-ACTRIIB) and mutants (HA-ACTRIIB-N42Q, -N65Q, and -N486Q) were constructed, followed by WB detection of the HA-tagged protein. Data are reported as means and SD (n=3). ***P<0.001; *t*-test and ANOVA.

PNGase F (peptide-N-glycosidase) and Endo H (endoglycosidase H) were used to verify if the high molecular weight bands were related to ACTRIIB N-glycosylation. PNGase F and Endo H were used to remove N-glycans in 5-8F and HNE2 cells. WB results showed that the ACTRIIB protein at the high molecular weight decreased after PNGase F and Endo H treatments (P<0.001; Figure 3A), while the protein at low molecular weight was increased (P<0.001). After PNGase F and Endo H treatment in NPC tissue samples, the ACTRIIB protein at the high molecular weight decreased or disappeared while the protein at the low molecular weight increased (P<0.001, Figure 3B). Tunicamycin (TM) is an inhibitor of N-glycosylation modification. After TM (100 ng/mL) treatment, the ACTRIIB protein at the high molecular weight decreased in 5-8F and HNE2 cells, while the protein at the low molecular weight increased (P<0.001; Figure 3C).

ACTRIIB wild type (WT, HA-ACTRIIB) and mutants (HA-ACTRIIB-N42Q, N65Q, and N486Q) were constructed. In the wild type, HA-tagged proteins were significantly more N-glycosylated, thus increasing the protein primarily at the high molecular weight, while the N42Q mutation led to the presence of the protein at low molecular weight (P<0.001; Figure 3D). N65Q and N486Q mutations did not affect the N-glycosylation-related modification of ACTRIIB, because ACTRIIB with N65Q and N486Q mutation was also at the high molecular weight similar to the wild type. This suggested that N-glycosylation modification is at N42 of ACTRIIB protein in NPC cells.

### N-glycosylation modification impacted the stability and location of ACTRIIB protein in NPC cells

**Figure 4:**
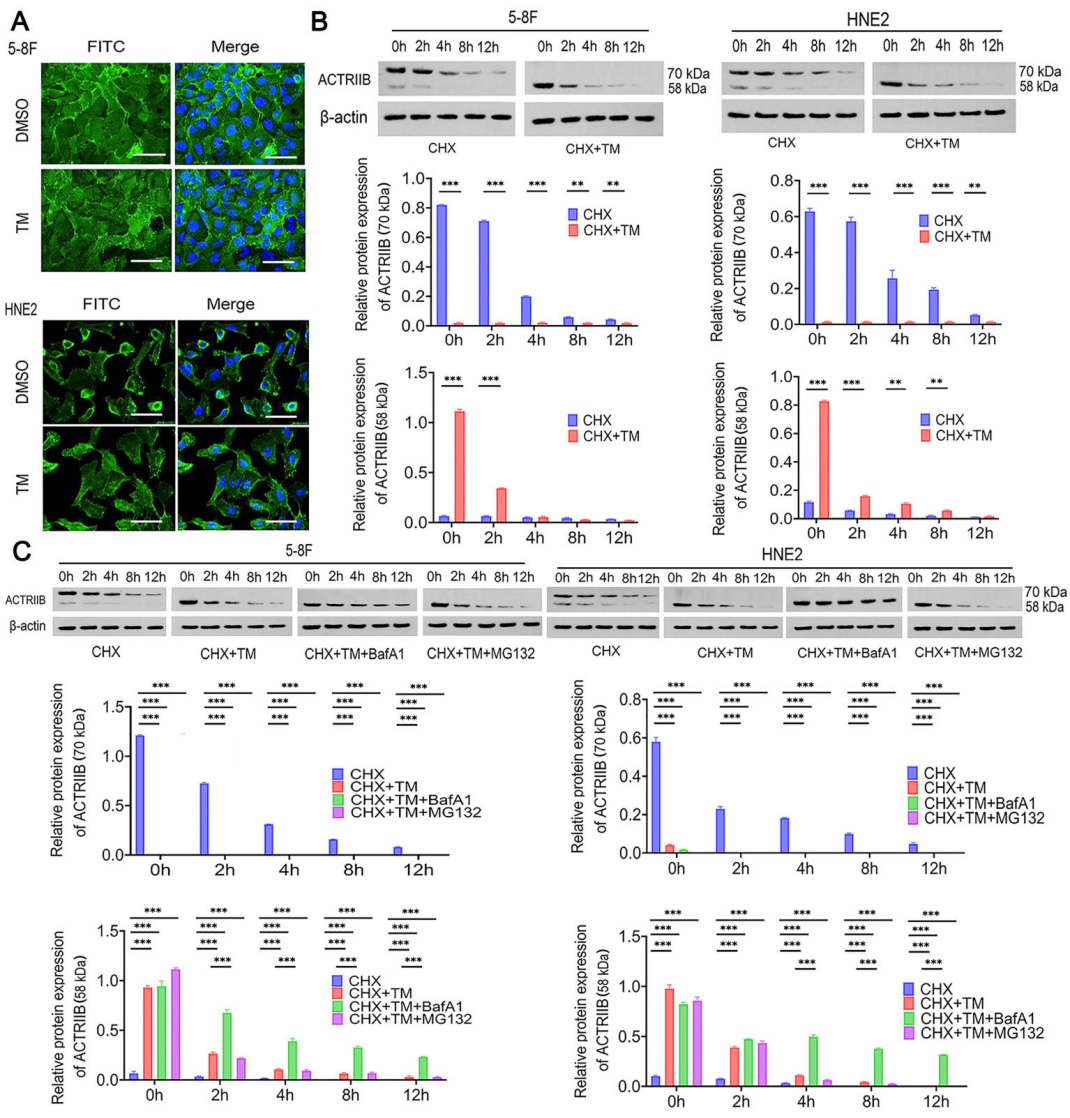
N-glycosylation modification impacted the stability and location of ACTRIIB protein in nasopharyngeal carcinoma (NPC) cells. **A**, Detection of ACTRIIB cellular localization by immunofluorescence after pre-treatment of cells with the N-glycosylation inhibitor tunicamycin (TM). Scale bars indicate 10 μm. **B**, Cells were pre-treated with the protein synthesis inhibitor cycloheximide (CHX). ACTRIIB protein gradually decreased at time points of 0, 2, 4, 8, 12 h after CHX treatment. Reduction in ACTRIIB N-glycosylation increased protein degradation, thereby suppressing protein stability. **C**, To understand how N-glycosylation influenced ACTRIIB protein stability, cells were treated with inhibitors of ubiquitin-proteasome system (MG132) and autophagy-lysosomes system (BafA1). Reduction in N-glycosylation increased lysosomal degradation of ACTRIIB protein. Data are reported as means and SD (n=3). **P<0.01 and ***P<0.001; ANOVA.

Immunofluorescence was conducted to detect the cellular localization of ACTRIIB. ACTRIIB was primarily localized at the cell membrane before treatment, while after TM treatment, it was mainly distributed in the cytoplasm. Therefore, ACTRIIB N-glycosylation was involved in the regulation of ACTRIIB membrane localization (Figure 4A). We further studied the effect of N-glycosylation on ACTRIIB protein stability. Cells were pre-treated with the protein synthesis inhibitor cycloheximide (CHX) to inhibit endogenous protein synthesis. ACTRIIB protein was gradually decreased at time points 0, 2, 4, 8, and 12 h after CHX treatment. Co-treatments with CHX and TM enhanced the reduction of ACTRIIB protein (P<0.01, Figure 4B). This indicated that the removal of N-glycosylation by TM leads to accelerated degradation of ACTRIIB protein, resulting in instability of ACTRIIB protein. To understand how N-glycosylation influenced ACTRIIB protein stability, the cells were treated with inhibitors of the ubiquitin-proteasome system (MG132) and autophagy-lysosomes system (BafA1). After CHX and TM treatment, ACTRIIB protein was primarily at low molecular weight and had faster degradation. The inhibitor BafA1 suppressed the ACTRIIB protein degradation after CHX and TM treatment (P<0.001; Figure 4C). However, MG132 had no such effect. This result suggested that N-glycosylation prevents the degradation of ACTRIIB protein by the autophagy-lysosomes system.

### Impact of ACTRIIB N-glycosylation on downstream targets and cell proliferation

**Figure 5:**
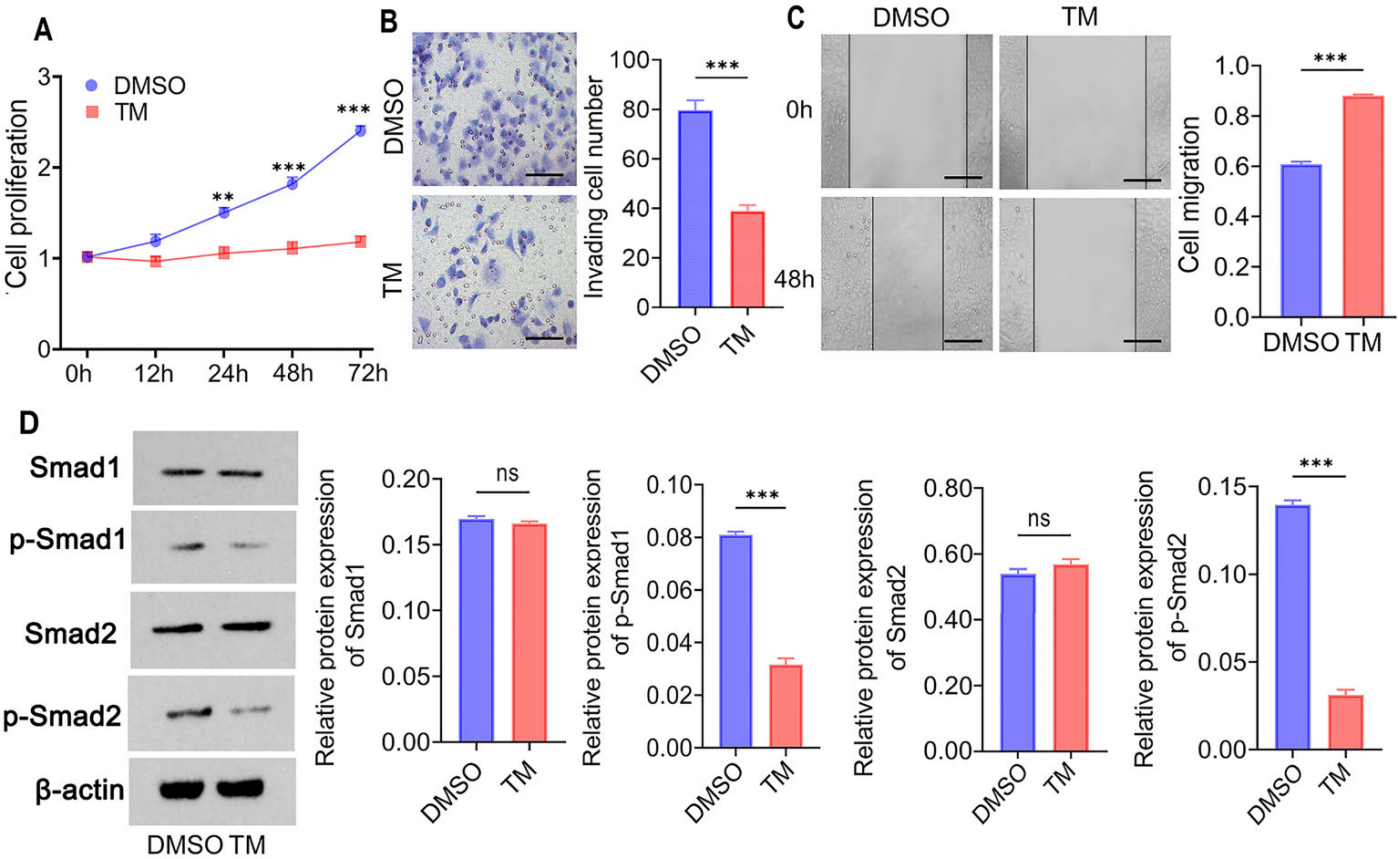
The impact of ACTRIIB N-glycosylation on downstream targets and cell proliferation. **A**, Tunicamycin (TM) treatment inhibited cell proliferation. **B**, TM treatment inhibited cell invasion capability. Scale bars indicate 40 μm. **C**, TM treatment inhibited cell scratch healing. Scale bars indicate 100 μm. **D**, WB detection of Smad1, phosphorylated Smad1 (p-Smad1), Smad2, and phosphorylated Smad2 (p-Smad2). Data are reported as means and SD (n=3). **P<0.01 and ***P<0.001; *t*-test and ANOVA. ns: non-significant.

5-8F cells were treated with TM for 48 h to assess cell proliferation, migration, and invasion. The results indicated that TM inhibited cell proliferation (P<0.01; Figure 5A), weakened invasion capabilities (P<0.001, Figure 5B), and reduced scratch healing (P<0.001, Figure 5C). WB was conducted to detect Smad1, p-Smad1, Smad2, and p-Smad2. TM reduced protein levels of p-Smad1 (P<0.001) and p-Smad2 (P<0.001, Figure 5D) without affecting the expression of Smad1 (P=0.361) and Smad2 (P=0.269). These data suggested that ACTRIIB N-glycosylation induces the activation of downstream Smad1/Smad2 and promotes cell proliferation, migration, and invasion.

### Knockdown of ACTRIIB or inhibition of N-glycosylation enhanced the sensitivity of NPC cells to docetaxel

**Figure 6:**
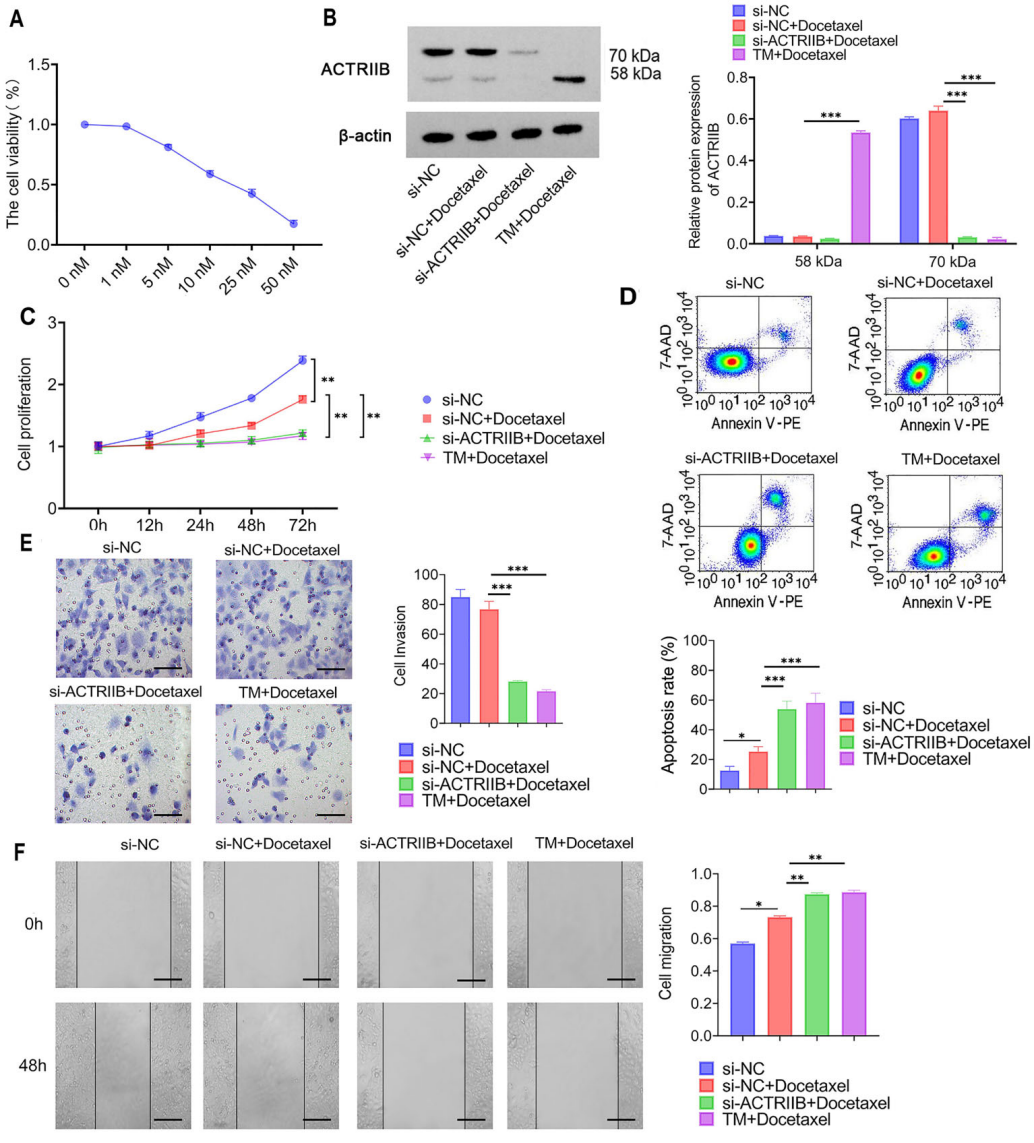
Knockdown of ACTRIIB or inhibition of N-glycosylation enhanced the sensitivity of nasopharyngeal carcinoma (NPC) cells to docetaxel. **A**, Determination of docetaxel drug sensitivity in 5-8F cells. **B**, WB analysis of ACTRIIB protein level in cells after transfection of si-NC or si-ACTRIIB and treatment with tunicamycin (TM) and docetaxel (5 nM). **C**, Detection of cell proliferation ability in cells after transfection of si-NC or si-ACTRIIB and treatment with TM and docetaxel (5 nM). **D**, Detection of apoptosis in cells after transfection of si-NC or si-ACTRIIB and treatment with TM and docetaxel (5 nM). **E**, Detection of cell invasion ability in cells after transfection of si-NC or si-ACTRIIB and treatment with TM and docetaxel (5 nM). Scale bars indicate 40 μm. **F**, Detection of cell scratch healing ability in cells after transfection of si-NC or si-ACTRIIB and treatment with TM and Docetaxel (5 nM). Scale bars indicate 100 μm. Data are reported as means and SD (n=3). *P<0.05, **P<0.01, and ***P<0.001; ANOVA.

The sensitivity of 5-8F cells to docetaxel was assessed by treating the cells with 0, 1, 5, 10, 25, and 50 nM docetaxel for 48 h. The IC20 was about 4-5 nM and the IC50 was about 15-20 nM. Subsequent experiments were performed with 5 nM docetaxel (Figure 6A). WB analysis of ACTRIIB showed that cell transfection of si-ACTRIIB with docetaxel (5 nM) treatment showed reduced protein levels at both molecular weights 58 kDa and 70 kDa. Co-treatment with TM and docetaxel (5 nM) showed a significant decrease in ACTRIIB protein at molecular weight 70 kDa (P<0.001, Figure 6B), but an increase in ACTRIIB protein at molecular weight 58 kDa (P<0.001). Cell proliferation, flow cytometry analysis of apoptosis, wound healing, and cell invasion assays showed that cell transfection of si-NC with docetaxel (5 nM) treatment inhibited cell proliferation (P=0.007, Figure 6C), cell invasion (P=0.066, Figure 6E), and wound healing ability (P=0.028, Figure 6F), with apoptosis increasing to 15-20% (P=0.046, Figure 6D), compared to control (si-NC). Compared to cell transfection of si-NC with docetaxel (5 nM) treatment, cell transfection of si-ACTRIIB with docetaxel (IC20) and the co-treatment with TM and docetaxel showed significant inhibition of cell proliferation (P<0.004, Figure 6C), cell invasion (P<0.001, Figure 6E), and wound healing ability (P<0.004, Figure 6F), with apoptosis increasing to over 30% (P<0.001 and P<0.001, Figure 6D). This indicated that inhibiting ACTRIIB expression or N-glycosylation significantly enhanced NPC cells' sensitivity to docetaxel.

## Discussion

This study unveiled the pivotal role of ACTRIIB's N-glycosylation in the proliferation and invasion of NPC cells, providing new insights into the involvement of ACTRIIB in the onset and development of NPC and offering potential targets for the development of novel therapeutic strategies. Our experimental results indicated that the N-glycosylation of ACTRIIB enhanced the protein stability and induced the membrane localization, thereby promoting the proliferation and invasion of tumor cells. Notably, we discovered that ACTRIIB's N-glycosylation occurred at a specific asparagine residue (N42), and that this glycosylation was crucial for the protein stability and the localization on the cell membrane.

Compared with previous studies, our findings further underscore the role of ACTRIIB in tumor development. Prior research has mainly focused on the signaling mechanisms of ACTRIIB and its role in the tumor microenvironment, while our study revealed a new mechanism of functional regulation of ACTRIIB from the perspective of post-translational modifications (7). N-glycosylation, a common post-translational modification, affects protein folding, stability, and cellular localization (23-25). Our research showed that N-glycosylation of ACTRIIB not only enhanced its membrane localization but also promoted the proliferation of tumor cells, consistent with findings in other types of tumors where ACTRIIB has been shown to promote tumor growth.

Furthermore, our study revealed that inhibiting the N-glycosylation of ACTRIIB can reduce the protein levels, decrease cellular proliferation, and enhance NPC cell sensitivity to docetaxel. These results provided important clues for developing new therapeutic strategies against nasopharyngeal cancer. Previous research has indicated the potential value of docetaxel in treating NPC (26), and our study further suggested that targeting ACTRIIB's N-glycosylation could enhance the efficacy of docetaxel, offering a potential combined therapy approach.

Although the study of the role of ACTRIIB's N-glycosylation in NPC provided valuable insights, it had some limitations in several aspects. Firstly, the discussion on the underlying molecular mechanisms was not sufficiently in-depth. Specifically, how ACTRIIB's N-glycosylation affects the TGF-β signaling pathway and its downstream effectors' activity, and how this process specifically influences tumor cell proliferation and invasion require further experimental clarification. Secondly, despite the experimental results indicating the key role of ACTRIIB's N-glycosylation in the proliferation of NPC cells, there was a lack of direct data from clinical samples to support the clinical relevance of these *in vitro* experimental results. Analysis of the relationship between ACTRIIB N-glycosylation levels and patient clinical characteristics (such as tumor staging, prognosis, etc.) would further strengthen the clinical significance of the study.

In summary, our research indicated the critical role of ACTRIIB's N-glycosylation in the proliferation and invasion of NPC cells and revealed a new mechanism by which inhibiting this modification process may enhance chemosensitivity. These findings not only provide a new perspective for understanding the pathogenesis of NPC, but also lay an important foundation for developing new treatment strategies. Future research should further explore the role of ACTRIIB's N-glycosylation in the development of NPC and how this mechanism can be effectively utilized for treatment.
